# Suppression of PDHX by microRNA-27b deregulates cell metabolism and promotes growth in breast cancer

**DOI:** 10.1186/s12943-018-0851-8

**Published:** 2018-07-16

**Authors:** Steven C. Eastlack, Shengli Dong, Cristina Ivan, Suresh K. Alahari

**Affiliations:** 1 Department of Biochemistry and Molecular Biology, Stanley S. Scott Cancer Center, LSUHSC School of Medicine, New Orleans, LA 70112 USA; 2 Department of Experimental Therapeutics, Division of Cancer Medicine, The University of Texas MD Anderson Cancer Center, Houston, TX 77030 USA

**Keywords:** Breast cancer, Metabolism, Warburg effect, microRNA, Lactate, Pyruvate dehydrogenase

## Abstract

**Background:**

The disruption of normal gene regulation due to microRNA dysfunction is a common event in cancer pathogenesis. MicroRNA-27b is an example of an oncogenic miRNA, and it is frequently upregulated in breast cancer. MicroRNAs have been found to deregulate tumor metabolism, which typically manifests as heightened cellular glucose uptake in consort with increased flux through glycolysis, followed by the preferential conversion of glycolytic pyruvate into lactate (a phenomenon known as the Warburg Effect). Pyruvate Dehydrogenase, an enzyme complex linking glycolysis with downstream oxidative metabolism, represents a key location where regulation of metabolism occurs; PDHX is a key structural component of this complex and is essential for its function.

**Methods:**

We sought to characterize the role of miR-27b in breast cancer by identifying novel transcripts under its control. We began by utilizing luciferase, RNA, and protein assays to establish PDHX as a novel target of miR-27b. We then tested whether miR-27b could alter metabolism using several metabolite assay kits and performed a seahorse analysis. We also examined how the altered metabolism might affect cell proliferation. Lastly, we confirmed the relevance of our findings in human breast tumor samples.

**Results:**

Our data indicate that Pyruvate Dehydrogenase Protein X is a credible target of miR-27b in breast cancer. Mechanistically, by suppressing PDHX, miR-27b altered levels of pyruvate, lactate and citrate, as well as reducing mitochondrial oxidation and promoting extracellular acidification. These changes corresponded with an increased capacity for cell proliferation. In human breast tumor samples, PDHX expression was deficient, and low levels of PDHX were associated with reduced patient survival.

**Conclusions:**

MicroRNA-27b targets PDHX, resulting in an altered metabolic configuration that is better suited to fuel biosynthetic processes and cell proliferation, thereby promoting breast cancer progression.

**Electronic supplementary material:**

The online version of this article (10.1186/s12943-018-0851-8) contains supplementary material, which is available to authorized users.

## Background

The past two decades have spawned a transformation in the understanding of gene expression and its regulation. This shift has been largely driven by the growing awareness of the profound role that noncoding gene products perform within cells, particularly microRNA (miRNA), which is a class of short (18-25 nt) regulatory RNAs first discovered in 1993 [1, 2]. As a group, miRNAs comprise roughly 1–2% of genes in mammals [3]. They participate in gene regulation via targeted silencing of specific mRNA transcripts by annealing to the 3’UTR and suppressing translation or initiating degradation of the transcript [4–7]. To this end, their impact on gene regulation is quite extensive, with some reports indicating that over 60% of protein coding genes harbor miRNA-binding sites [8].

Given their widespread involvement in control of gene expression, dysfunction of normal miRNA regulation has emerged as an important mechanism in disease, especially cancer. One such example is miRNA-27b, which primarily functions as an oncogenic miRNA (oncomir) in breast cancer (BC) by targeting multiple tumor-suppressor genes. For example, we have previously reported that miR-27b suppresses Nischarin and ST14—both tumor suppressor proteins involved in migration and invasion [9, 10]. Others have described a role in promoting tumor growth and metastasis by targeting HIC1 [11] and PSAP [12]. Thus, miR-27b appears to perform a multi-faceted role in cancer promotion, regulating multiple different oncogenic processes.

In considering this pattern, we wondered what other oncogenic cell processes miR-27b may promote in BC. In this study, we report for the first time evidence linking miR-27b overexpression in cancer to the deregulation of energy metabolism—an emerging hallmark of cancer [13–15]. Specifically, we show the mechanism for these metabolic effects is due to miR-27b targeting of Pyruvate Dehydrogenase Protein X (PDHX), a structural component of the PDH complex. Although it does not have catalytic function, PDHX is required for the activity of the enzyme complex [16]. In the mitochondria, the PDH complex catalyzes the oxidative decarboxylation of pyruvate into an acetyl group attached to a Coenzyme A molecule. Because acetyl-CoA can subsequently be condensed with oxaloacetate to generate citrate, PDH is positioned at the intersection of glycolysis and the citric acid cycle, an essential conduit between the oxygen-independent and dependent pathways. Given the fundamental importance of this metabolic linkage, PDH deficiency leads to disastrous consequences [17]. Nevertheless, the selective inhibition of this complex and the metabolic alterations which ensue appears to be an early event within the pathogenesis of cancer [18, 19]. Therefore, the loss of function and/or expression of the PDH complex components represent a common mechanism for cells in transition towards a pro-glycolytic configuration. Repression of the PDHX subunit in particular has been linked to the loss of overall PDH complex functioning and resulting deregulation of glucose metabolism in colorectal cancer [20]. Given such reports, we make the case that PDHX (and the PDH complex in general) effectively functions as a tumor suppressor by maintaining normal metabolic homeostasis.

The propensity of tumor cells to shift away from aerobic metabolism is a well-documented phenomenon tracing back nearly a century to Otto Warburg’s seminal observation that tumor tissues display characteristic shifts in energy metabolism—a phenomenon referred to as the Warburg Effect [21]. Tumor cells uptake glucose rapidly, increase glycolytic flux, and oxidize a reduced fraction of pyruvate, which is fermented into lactate instead. Early attempts to explain this feature proposed that mitochondrial dysfunction was responsible [22]. However, it has since become clear that mitochondrial function remains intact in transformed cells and in fact is critical for cancer cell function [23–25]. This is largely because numerous and essential reactions of macromolecule biosynthesis occur within and are necessary for cell proliferation. We hypothesize that miR-27b facilitates this shift through the following mechanism: by targeting PDHX, miR-27b reduces its expression and therefore the function of the PDH complex, resulting in reduced oxidation of pyruvate, subsequently reducing mitochondrial respiration and altering levels of several metabolites, including pyruvate, lactate, and citrate. Our experiments indicate that this effect occurs only in the context of cancers that highly overexpress miR-27b; cancer cells with low/normal miR-27b expression preserve PDH function. Below, we describe a series of experiments leading us to conclude that PDHX is a functional target of miR-27b and that this interaction has consequential effects on cell metabolism which facilitate cell growth and progression in breast cancer.

## Methods

### MicroRNA target prediction

The miRWalk target prediction algorithm was utilized to identify putative miR-27b targets [26]. In addition to providing its own predictions, miRWalk also houses a composite of several additional prediction algorithms. In our assessment, DIANAmT, miRanda, miRDB, RNAhybrid, PICTAR, PITA, RNA22, and TargetScan algorithms were also used. A cut-off was set for each program giving a binary prediction indicated as 1 or 0. Only those targets predicted by at least eight out of nine algorithms were selected for subsequent experimental validation.

### Cell culture and reagents

HEK-293T cells and human breast cancer cell lines MCF7 and MDA-MB-231-4175 (a gift from Dr. Joan Massague, Memorial Sloan-Kettering Cancer Center, New York) were maintained in Dulbecco’s modified Eagle’s medium (DMEM) supplemented with 10% fetal bovine serum. ZR75 cells (ATCC) were cultured in RPMI 1640 with 10% fetal bovine serum. Stably transfected ZR75 cells (expressing miR-27b or scr) and 4175 (expressing miR-27b-ZIP or scr) were created by our lab previously [9].

### Transfection of miR-27b mimic and anti-miR-27b oligonucleotides

MicroRNA-27b mimic and antisense oligomer (ASO) were purchased from Switchgear Genomics. The day before transfection, 1.5 × 10^5^ cells (4175 or MCF7) were seeded onto 6-well plates. A final concentration of 5 μM of mimic, ASO, and scramble control were transfected using Opti-MEM and Lipofectamine 2000 as per the manufacturer’s specifications (Invitrogen). In co-transfections with expression vectors, we used 1 μg DNA per 3.5 cm well. With this approach, we routinely achieve ~ 70% transfection efficiency in MCF7 cells and ~ 50% in 4175 cells. Protein and mRNA were collected 48 h after transfection.

### 3′-UTR luciferase reporter constructs and luciferase assays

We obtained ten different GoClone 3′UTR luciferase reporter constructs from Switchgear Genomics and cotransfected each with miR-27b mimic, ASO, or NTC as described above. After 24 h, the luciferase activity was evaluated using the LightSwitch Luciferase Assay kit (Switchgear Genomics) according to manufacturer protocol. Luminescence was measured using a Lumat3 single-channel luminometer (Berthold Technologies). To create the non-targetable mutant vector, the original PDHX expression vector was mutated using overlap extension PCR. All 8 of the nucleotides corresponding to the seed region of miR-27b were mutated to ensure loss of affinity for the 3’UTR, by miR-27b. The following primers were used in the PCR experiment: 5’-TGAGGGCCCTCCTTCTAAAGCAAGAGGATAAAAGAAGC-3′ (SeedMuFwd) and 5′- TGAGGGCCCGTTATATAAGTGAAAAACAAGACAGATAGAAACC-3′ (SeedMuRvs). The inserted sequence replacing the original eight bases is derived from the ApaI site (GGGCCC) and trinucleotide overhang (TGA) located on the 5’end of each primer.

### Normal and tumor breast tissues

Fifty-two normal breast tissues and fifty-four breast tumors tissues were obtained as surgical samples from patients throughout the US. Frozen tissue sections were obtained from five divisions of the Cooperative Human Tissue Network: southern (Birmingham, AL), eastern (Philadelphia, PA), mid-Atlantic (Charlottesville, VA), Midwestern (Columbus, OH), and western (Nashville, TN).

### Western blotting

Cells were washed twice with cold phosphate-buffered saline and lysates were prepared in 350 μl of radioimmunoprecipitation (RIPA) lysis buffer (50 mM Tris-HCl pH 7.5, 150 mM NaCl, 50 mM NaF, 1% NP-40, 0.1% sodium deoxycholate, 1 mM sodium pyrophosphate, 1 mM Na_3_VO_3_ and Roche protease inhibitor cocktail) and centrifuged at 16,000 *g* for 20 min at 4 °C. 6X SDS sample buffer was added to each sample prior to boiling for 15 min and all were stored at − 80 °C until analysis. Small aliquots (10 μl) of the lysates were used for protein determination with a BCA protein assay according to manufacturer protocols (Bio-Rad). Protein samples (20–50 μg) were separated by SDS-PAGE in 9% gels and transferred onto polyvinylidene difluoride membranes (GE Healthcare). The membranes were blocked in 5% milk in 0.1% Tris-buffered saline-Tween 20 for 1 h at room temperature. Afterwards, membranes were incubated with PDHX or Vinculin primary antibodies (Santa Cruz Biotechnology) either overnight at 4 °C or for 2 h at RT. Antibody binding was revealed by incubation with horseradish peroxidase-conjugated secondary antibodies (Santa Cruz Biotechnology) and an ECL Plus immunoblotting detection system (GE Healthcare). For measurement of PDHX protein levels in tumor samples, 0.5-1 mg pieces of breast tumor and pair-wise matched normal breast tissue were used. Briefly, the samples were submerged in liquid N2 and pulverized into a fine powder using a mortar and pestle. This was suspended in RIPA lysis buffer at a concentration of 100 mg/ml and sonicated. Tissue lysates were subsequently processed in the same manner as the cell lysates described above. 10-20 μL of sample per well was used for the electrophoresis and PDHX protein was detected by Westernblotting.

### Reverse transcription and real time PCR of PDHX and miR-27b

Expression of mature miRNAs was quantitated using TaqMan microRNA assays (Applied Biosystems) specific for miR-27b. Each sample was analyzed in triplicate. Reverse transcription was performed using the TaqMan MicroRNA Reverse Transcription Kit (Applied Biosystems), 10 ng of total RNA input, and TaqMan looped RT primers specific for miR-27b or RNU6B control. Real time PCR was performed using standard TaqMan protocols on a LightCycler480 Instrument (Roche). The 20-μl PCR reactions included 1.33 μl of RT product, 10 μl of TaqMan Universal PCR Master Mix, No AmpErase UNG (Applied Biosystems), and 1 μl of primer and probe mix (Applied Biosystems). The reactions were incubated in a 96-well plate at 95 °C for 10 min, followed by 40 cycles of 95 °C for 15 s and 60 °C for 1 min. The level of miRNA expression was measured using *Ct* (threshold cycle). The Δ*Ct* was calculated by subtracting the *Ct*_U6_ from the *Ct*_miR-27b_. The ΔΔ*Ct* was calculated by subtracting the Δ*Ct* of the control cells from the Δ*Ct* of the experimental cells. Fold change was generated using the 2^−ΔΔCt^ equation.

PDHX expression was examined in cell line samples as well as in human breast normal and tumor tissues. cDNAs were synthesized from 1 μg of tumor RNA using the high capacity cDNA reverse transcription kit (Applied Biosystems). This cDNA was used for both qPCR and conventional PCR experiments. GAPDH was used as a loading control. Primers for GAPDH are described previously [27]. PDHX primers were designed using Primer3. Their sequences are as follows: Fwd: 5′-AAG ATT ACC GAC TCC AGA CCA A-3′ and Rvs: 5′-TGT CCA GGA GTT GAT ACT GCT G-3.’ Reactions were performed in triplicate on a benchtop thermal cycler in the following conditions: 30 cycles at 95 °C for 15 s, and 60 °C for 30 s, and 68 °C for 1 min. PCR products were electrophoresed on a 1% agarose ethidium bromide gel for 1 h at 75 V and imaged using a ChemiDoc imager (BioRad). For quantitative PCRs, each 20-μl PCR reaction volume included 2 μl of RT product, 1 μl primers, and 10 μl of SYBR Green I Master mix (Roche). The reactions were incubated in a 96 or 384-well plate at 95 °C for 10 min, followed by 40 cycles of 95 °C for 15 s and 60 °C for 1 min. qPCRs were performed using a LightCycler480 Instrument (Roche). Human GAPDH was used as the housekeeping control to normalize the PDHX expression data by the ΔΔ*Ct* method outlined above.

### Metabolite level measurement and PDH activity assays

For extracellular lactate, pyruvate and citrate measurements, complete medium was collected 24 h after plating 1.5 × 10^5^ cells/well in 6-well plates. The medium was centrifuged to remove and cell debris and diluted 1:10 in fresh DMEM. Lactate, citrate, and pyruvate levels were assessed in 10ul of medium using the EnzyChrom kits designed to measure of the three metabolites according to manufacturer protocol (BioAssay Systems). Results were normalized to total cell protein by BCA assay or by counting cells in the sample following collection of medium. Lactate and assays were read at 565 nm using colorimetric micro-plate reader (BioTek); pyruvate and citrate assays were read using a fluorometric micro-plate reader at 535 nm excitation 590 nm emission. For intracellular lactate, cells were collected in RIPA lysis buffer as described above. 10ul of this lysate was used in place of DMEM and RIPA was used for blanks and all dilutions for the lactate standard curve. Intracellular pyruvate and citrate were measured by collecting cells and resuspending them in PBS, followed by sonication to lyse the samples. Measurement of overall PDH complex activity was performed using the Pyruvate Dehydrogenase Assay Kit according to manufacturer protocols (BMR, Buffalo, NY). Assays were read using a colorimetric micro-plate reader at 565 nm (BioTek). Results were normalized to total protein level measured by BCA assay and graphed as a percent of PDH activity in the scramble control cells.

### Generation of stable PDHX shRNA cells

HEK-293 T cells were transfected with 10 μg of one of four unique PDHX shRNA or scramble control Lentivectors purchased from Applied Biological Materials (Richmond, BC). Target sequences of shRNA we used are as follows: sh1: 3’-AGAGTTATTGCCAGAGATTAACTGAATC-5′; sh2: 3’-GCTGTTACCCTTAAACAAATGCCAGATGT-5′ sh3: 3’-AGTCACAATGTCAAGTGACAGTCGAGTGG-5’ shScr: 3’-GGGTGAACTCACGTCAGAA-5.’ Each dish was also cotransfected with ABM’s Third Generation lentiviral packaging mix, using lipofectatmine 2000 and Opti-MEM as described above. Medium containing lentiviral particles was collected after 24 h, replaced with fresh medium, followed by a second collection after 24 additional hours. The two collections were pooled together and then ultracentrifuged to concentrate the particles. The pellet was resuspended in 100ul PBS and 25ul of this was added with polybrene to MCF7 and ZR75 cells in 6-well plates and incubated overnight. The viral medium was replaced with fresh complete medium the following day. After 1 week, we used a puromycin selection (1μg/mL) to eliminate any cells which did not successfully incorporate the shRNA lentivector. Furthermore, microscopy confirmed the surviving cells to be > 95% GFP positive.

### Seahorse XF-24 metabolic flux analysis

Oxygen Consumption rate (OCR) and extracellular acidification rate (ECAR) in MCF7, ZR75, and 4175 cells were measured at 37 °C using the Seahorse 24XF instrument (Agilent, Santa Clara, CA) as previously described [28]. Briefly, cells were seeded in a 24-well tissue culture plate at a density of 40,000 cells per well for 12 h. Cells were then changed to unbuffered XF assay media at pH 7.4 supplemented with 25 mM glucose and 1 mM sodium pyruvate. After cells incubated for 1 h at 37 °C in a non-CO_2_ incubator, respiration was measured before and after the injection of three compounds: oligomycin (1.5 μM), carbonyl cyanide 4-(trifluoromethoxy) phenylhydrazone (FCCP) (2.5 μM), and rotenone (1 μM) at injection 3. Experiments were performed in real time in six replicate wells for each cell line. OCR and ECAR were automatically calculated by the Seahorse XF-24 software. Immediately following each run, the cells were lysed in RIPA buffer and the protein concentration was determined by BCA assay for normalization.

### MTT and colony formation assays

Cell proliferation was assessed using a 3-(4,5-dimethylthiazol-2-yl)-2,5-diphenyltetrazolium bromide (MTT) assay. Stably transfected cells were seeded at 5000 cells/well in 96 well plates and incubated for 1–4 days, after which 10 μl of 5 mg/ml MTT (Cayman Chemical) was added to each well. The wells were then incubated at 37 °C for 3.5 h. The purple-blue MTT formazan precipitate was dissolved in 150 μl of MTT solvent (4 mM HCl, 0.1% NP-40 in isopropanol). Increases in cell number result in greater amounts of formazan production and thus increased optical density, measured at 562 nm in a micro-plate reader (Bio-Rad). Since a uniform cell number was plated initially, increased absorbance in one sample compared to another indicating a greater rate of cell proliferation was present. For colony formation assays, cells were seeded at very low density (5000 cells total per 3.5 cm dish) and incubated for 10 days to allow for individual cells to form macroscopic colonies with regular replacement of overlying medium. After the incubation, cells were washed with PBS, fixed in 4% paraformaldehyde and stained with crystal violet. Images of each well were captured and used for quantitation of growth by counting of colony numbers in a standardized visual field for each well.

### Statistical analyses and datamining

Results are expressed as means ± SEM. In making comparisons between two groups for statistical analyses, we used two-tailed nonpaired *t*-test (GraphPad Prism, version 5). Differences with *p* values of < 0.05 were considered significant. The Oncomine platform (Oncomine.org) was used to access and analyze data from the Cancer Genome Atlas (TCGA), Curtis Breast Cancer Database, and two cell line datasets (Scherf and Staunton Cell Line Statistics). Images of summary output data were captured and shown as they appeared in the Oncomine interface. The BioExpress database (hive.biochemistry.gwu.edu) was used to assess PDHX gene under-expression across several types of cancer. For XY correlation plots of PDHX and miR-27b in breast cancer, TCGA data was used. For correlation plots in normal tissues, we retrieved PDHX, miR-27b, and C9orf3 expression data from the GTEx portal database (gtexportal.org). The expression level of each individual tissue type was plotted as a single data point using GraphPad. spearman correlation r was calculated for the XY pairs in the plot and fitted with a trend line to display the correlation.

For correlation studies in breast cancer samples, statistical analyses of TCGA data were performed in R (version 3.4.1) (http:///www.r-project.org/). The statistical significance was defined as a *p*-value less than 0.05. We downloaded patient clinical information for the TCGA patients with breast invasive carcinoma (BRCA) from cBioPortal (http://www.cbioportal.org/). For the miRNA-Seq data in primary tumors, we derived the ‘reads_per_million_miRNA_mapped’ values for the mature form hsa-miR-27b (MIMAT0000419) from the Isoform Expression Quantification files in the Genomic Data Commons Data Portal (https://portal.gdc.cancer.gov/). PDHX gene expression quantification data for primary tumors was downloaded from Genomic Data Commons Data Portal in order to retrieve FPKM files. Log2-transformation was applied to the both miRNASeq and mRNASeq data. We ended up with 834 cases in total that had miRNA data, mRNA data and clinical information available. The Pearson correlation test was applied to measure the strength of the association between PDHX expression and miR-27b expression.

For survival analysis a Univariate Cox proportional hazards model was fitted to evaluate the association between overall survival (OS), respectively disease free survival (DFS) and covariates including PDHX expression levels and available clinical variables (age at diagnosis, stage). Stage, and PDHX level were statistically significant factors in the univariate Cox proportional hazards models for ER- cases, and were included in the final multivariable analysis of overall, and disease free survival. PDHX was an independent predictor for good prognosis in both cases (OS: HR = 0.42, CI(95%) = (0.22, 0.84), p-val(Wald) = 0.0132; DFS: HR = 0.32, CI(95%) = (0.14, 0.74), p-val(Wald) = 0.0079). In order to visualize the survival difference we used the log-rank test to find the point (cut-off) with the most significant (lowest *p*-value) split in high vs low mRNA level groups. The Kaplan-Meier plots were generated for these cutoffs (0.13 for OS; 0.24 for DFS).

## Results

### MicroRNA-27b targets the 3’UTR of PDHX

**Fig. 1:**
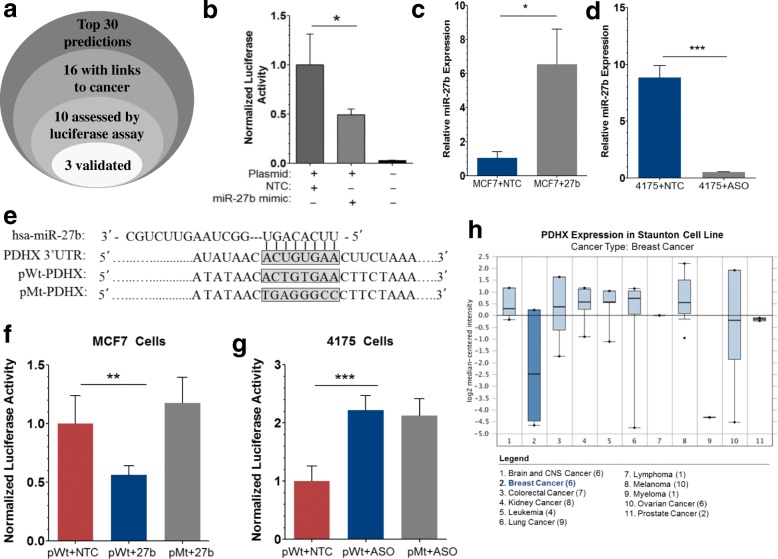
In silico and experimental evidence predict the 3’UTR of PDHX is targeted by MicroRNA-27b. **a** Diagram illustrating the stepwise approach used to select potential miR-27b targets for experimental validation. **b** Preliminary luciferase assay of MCF7 cells transiently cotransfected with a PDHX luciferase vector and miR-27b mimic or control. Luciferase expression is quantitated by measuring the degree of luminescence detected by a luminometer 24 h post-transfection for each assay. **c** and **d** TaqMan qRT-PCR assessing miR-27b levels in transfected MCF7 and 4175 cells to confirm adequate delivery of RNA oligonucleotides into the cell. **e** Nucleotide sequences indicating the location of miR-27b binding within the PDHX 3’UTR. The sequence of the original luciferase construct and the eight base pair substitutions present in the mutant construct are shown beneath. Luciferase assays in MCF7 (**f**) and 4175 (**g**) cells, showing miR-27b targeting of the PDHX-3’UTR sequence is lost upon mutation of the seed region. **h** Expression of PDHX in the Staunton Cell line dataset accessed online using Oncomine. PDHX Fold Change (vs average of all cancers): − 5.602; *p*-value: 0.023. Under-expression Gene Rank for PDHX: 335 (in top 7%)

There are multiple miRNA target prediction programs available for identifying putative targets of a miRNA of interest, each providing comparable but non-identical predictions depending on how the various parameters are weighted. However, it is not clear which one of these is the best [29]. Thus, we opted to use the miRNA target prediction program miRWalk in our initial screen for miR-27b targets. In addition to offering its own predictions, miRWalk also houses a composite of several other prediction algorithms (including miRanda, PITA, RNA22, picTar, and TargetScan) providing a comparative analysis of predicted targets. Using this consensus approach, we compiled the top thirty best candidates and selected from among them those with reported involvement in cancer, narrowing the list to sixteen. We picked ten of these in silico predictions for in vitro validation by luciferase assay. The initial luciferase assays were performed in MCF7 cells transiently cotransfected with a plasmid containing the luciferase gene cloned upstream of the target 3’UTR, along with a miR-27b mimic or non-targeting control. Compared to control MCF7 cells, miR-27b mimic produced a strong induction of luminescence, a proxy for the level of luciferase in the sample. Of the ten tested, three were found to be miR-27b targets in the initial luciferase screen: PDHX, PLK2, and PPP1CC (Fig. 1a, b and Additional file 1: Figure S1a, b). Of note, each of these three targets was predicted to be a target by at least 8 of the 9 algorithms (Additional file 2: Table S1). The seven others did not show a significant reduction in luminescence in thus do not appear to be regulated by this miRNA (Additional file 1: Figure S2). Thus, we selected PDHX as the primary target to investigate further because of its significance in cell energy metabolism, an area of growing interest in cancer biology. It should be noted that accuracy of these luciferase assays is dependent upon the success of the initial transient transfection. Using MCF7 cells we routinely achieve ~ 70% transfection efficiency. However, as an added measure to confirm transfection success, the levels of intracellular miR-27b were measured directly by qRT-PCR 24 h post-transfection (Fig. 1c).

To further validate PDHX targeting, a second set of luciferase assays was performed in which we used 4175 cells, a derivative of the MDA-MB-231 cell line in addition to MCF7 cells as before. Previous evidence published by our lab measuring miR-27b across a panel of BC cell lines indicated that MCF7 cells display low basal expression of miR-27b while in 4175 cells it is significantly elevated [10]. Thus, we opted to to suppress the expression of miR-27b with an antisense miR-27b oligonucleotide (ASO) in 4175 cells that overexpress endogenous miR-27b. Transfection efficiency of ASO in 4175 cells was also confirmed by qPCR (Fig. 1d). Along with the original PDHX luciferase vector containing a wild type 3’UTR (pWt), we generated a non-targetable mutant construct (pMt) harboring mutations at all eight nucleotides in the seed region of the PDHX 3’UTR (corresponding to nucleotides 1–8 on the 5′ end of the miRNA) (Fig. 1e). Perfect complementarity in this region is critical for miRNA:mRNA hybridization; mutating even one base pair can dramatically reduce miRNA targeting of the transcript. To ensure complete loss of fidelity, all eight nucleotides were mutated.

In MCF7 cells, miR-27b co-transfection reduced overall luminescence; however, as expected, no significant difference from the control was observed when the mutant construct was used, as miR-27b should no longer have affinity for vector (Fig. 1f). In 4175 cells, ASO transfection led to a restoration of wild type luciferase expression, suggesting that the ASO reduced levels of endogenous miR-27b sufficiently to reinstate luciferase expression (Fig. 1g). As expected, expression of the pMt luciferase did not benefit from ASO cotransfection because its mutant 3’UTR is not susceptible to miR-27b targeting. To compare the significance of PDHX suppression in breast cancer specifically when compared with other cancer types, we utilized the Staunton Cell Line Statistics database (accessed through the Oncomine data portal) to compare PDHX expression across a panel of different types of cancer cell lines. Interestingly, of the 11 different types of cancer available for comparison, breast cancer cells had among the lowest expression of PDHX, second only to myeloma (Fig. 1h). Another cancer cell line dataset (Scherf Cell Line database) showed similar findings (Additional file 1: Figure S3). Moreover, gene expression of PDHX using the BioExpress curated gene database revealed that PDHX is underexpressed in nearly 60% of breast cancer tumors (Additional file 1: Figure S4). These data suggest the suppression of PDHX is particularly important in the case of BC when compared to other malignancies.

### PDHX mRNA and protein expression are suppressed by miR-27b in vitro

**Fig. 2:**
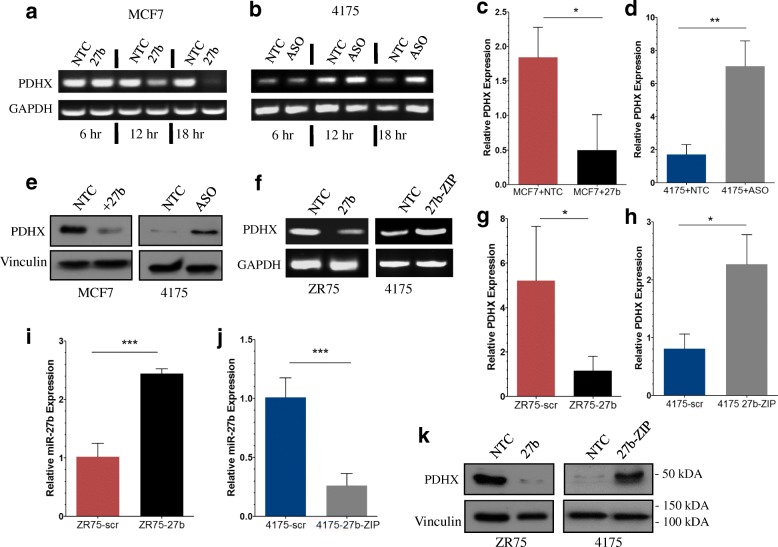
PDHX mRNA and protein expression is suppressed by miR-27b in vitro. **a** and **b** Time course RT-PCR of cells transiently transfected with the indicated oligonucleotide. **c** and **d** RNA was collected from cells as above for evaluation of PDHX expression by quantitative RT-PCR using SYBR Green to probe for PDHX expression. **e** Cell lysates from transiently-transfected MCF7 and 4175 cells were collected and probed for PDHX protein and Vinculin loading control. ZR75 cells stably expressing elevated miR-27b or scr control, as well as 4175 cells expressing either the 27b-ZIP construct or scr control were used to replicate the experiments in transiently transfected cells. RNA was also collected from the stable cell and reverse transcribed for quantitation of PDHX expression by standard PCR followed by gel electrophoresis (**f**) as well as qPCR (**g** and **h**). Confirmation of the miR-27b expression status of the ZR75 (**i**) and 4175 (**j**) stable cell lines (previously generated in our lab) was completed by TaqMan qRT-PCR. The same RNA samples collected for PDHX qPCR were again used to measure the miR-27b level of these cells. **k** IB of PDHX protein using stable cell lysates as in panel **e**

Encouraged by these preliminary findings, we proceeded to test whether PDHX mRNA expression was suppressed by miR-27b. We designed a time course experiment in which RNA was collected over a range of time points post-transfection for analysis by RT-PCR and gel electrophoresis. At 6 h post-transfection no change in PDHX expression was detected, but at 12 and 18 h, the intensity was increasingly reduced in the miR-27b-transfected MCF7 cells, indicating destabilization of PDHX transcripts by miR-27b (Fig. 2a). Similarly, by 18 h ASO transfection in 4175 cells increased PDHX expression (Fig. 2b). We reproduced this finding by PCR (Fig. 2c, d). Compared to control cells, PDHX expression was reduced in MCF7 cells transfected with miR-27b and increased in 4175 cells transfected with miR-27b ASO.

Ultimately, for miRNA-mediated suppression of PDHX mRNA to have a functional consequence in the cell, it must lead to reduced levels of the functional product of the gene. Thus, we next assessed protein levels of PDHX in transiently transfected cells by western blotting. Analogous to the changes in mRNA levels found prior, we observed a decrease upon transfection of miR-27b mimic and increase with ASO (Fig. 2e).

We also tested whether miR-27b targeting of PDHX could be reproduced in cells with stable miR-27b expression. ZR75 BC cells stably expressing miR-27b or non-targeting control (NTC) and 4175 cells expressing an anti-miR-27b “zipper” construct (miR-27b-ZIP) or control (previously created by our lab) were assessed by RT-PCR for PDHX expression detected with gel electrophoresis (Fig. 2f). Once again, miR-27b reduced PDHX mRNA in ZR75 cells and miR-27b knockdown in 4175-27b-ZIP cells increased its expression. Similarly, quantitation of PDHX in these cells by qRT-PCR revealed lower PDHX expression in ZR75-27b cells versus control and greater PDHX expression in 4175-27b-ZIP cells versus control (Fig. 2g, h). To confirm the stable transfection of these cells, we performed another TaqMan qPCR for miR-27b levels which confirmed the miR-27b expression status of these cell lines (Fig. 2i, j). Finally, we collected protein lysates from these cells and probed for PDHX protein. As expected, the results revealed an inverse correlation between miR-27b expression and PDHX protein levels (Fig. 2k).

### MicroRNA-27b overexpression alters metabolite levels while suppressing PDH complex activity

**Fig. 3:**
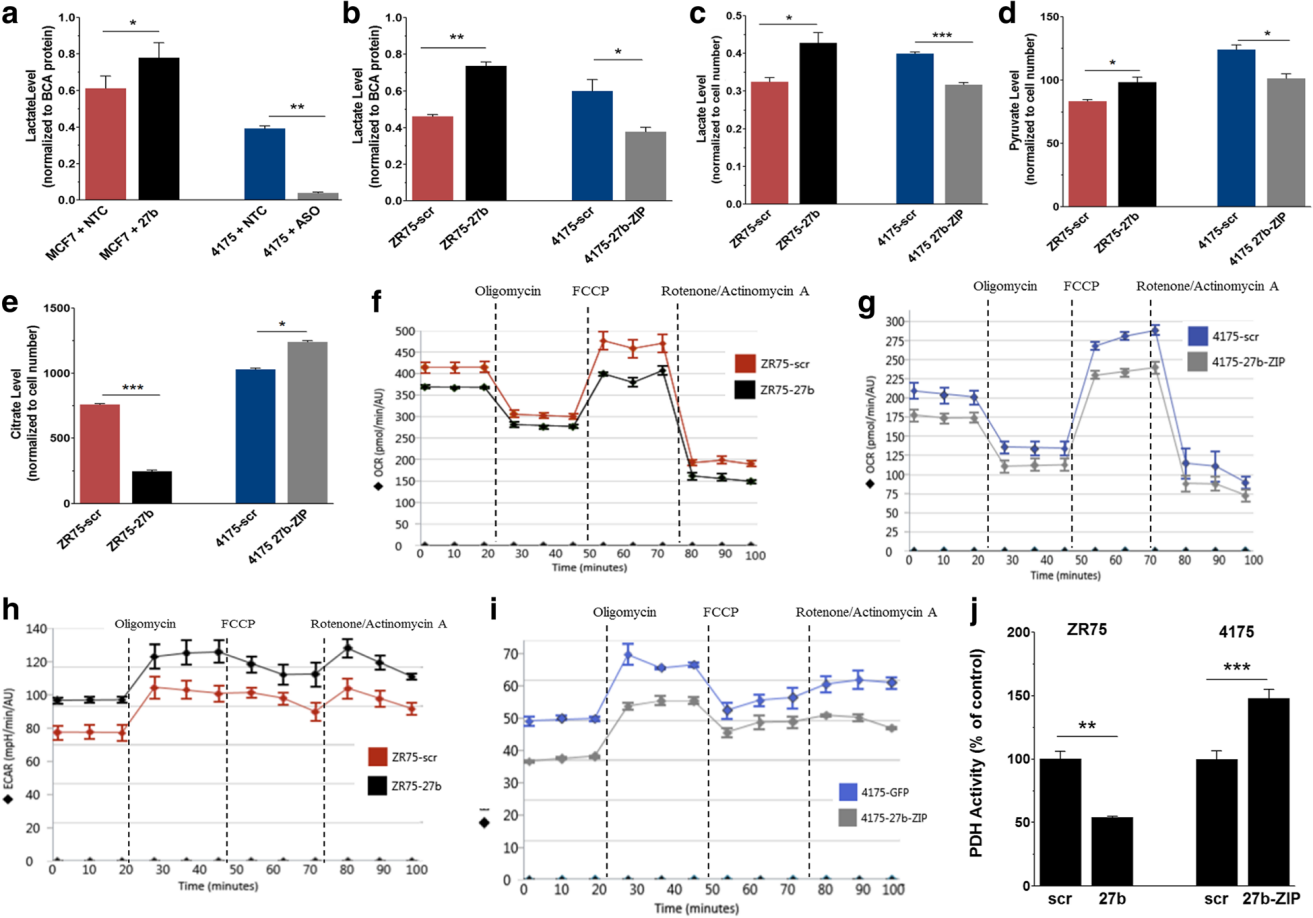
MicroRNA-27b overexpression alters metabolite levels while suppressing PDH complex activity. **a** MCF7 and 4175 cells were plated and transiently transfected with the indicated oligonucleotides. After 24 h the overlying growth medium was collected and analyzed to determine lactate concentration. **b** Lactate assays were repeated in stably transfected cells in the same manner as in panel **a**. Additionally, lysates of the underlying cells were collected and the intracellular lactate (**c**). These data were normalized to the cell protein level determined by BCA assay. Lysates were also collected for pyruvate (**d**) and citrate (**e**) assays. Seahorse mitochondrial stress test quantitating OCR and ECAR in ZR75 (**f** and **h**) and 4175 cells (**g** and **i**) in response to miR-27b expression levels. Results are normalized to BCA protein of the cells in each well which were collected after each assay (**j**) Cell lysates were probed for global PDH complex activity in the samples a PDH complex activity assay kit. All PDH activity and extracellular metabolite data are normalized to the total cell protein in each well by BCA assay. Data from intracellular metabolite assays (lactate, pyruvate and citrate) was normalized to the cell number counted immediately prior to collection

To confirm that the alterations in PDHX mRNA and protein levels are biologically consequential for the cell, we assessed for changes in cell metabolism. Firstly, we measured lactate levels in transiently and stably transfected cell lines. Since conversion into lactate is an alternative path for glycolytic pyruvate (instead of decarboxylation into acetyl-CoA by PDH), we hypothesized that a reduction in PDH activity would lead to greater lactate production as accumulating pyruvate is diverted into alternative pathways instead. Export of tumor-derived lactate is primarily mediated by the Monocarboxylate transporters (MCTs), which are frequently overexpressed in cancer [30, 31]. Thus, we measured both intracellular and extracellular lactate level. We uniformly plated each cell line and cultured them in standard growth medium for 24 h. Overlying medium was collected and its lactate content measured using an EnzyChrom lactate assay kit. In both our transient and stable expression cell models, increased miR-27b positively correlated with lactate production (Fig. 3a, b). We also measured intracellular lactate in the stable cell lines and observed the same result (Fig. 3c). These findings support the notion that miR-27b targets PDHX, since a reduction in overall PDH complex functioning would result in the accumulation of its substrate (pyruvate), some of which would consequently be shunted into lactate production.

To support this interpretation, we measured cellular pyruvate, and found it was also increased as expected (Fig. 3d). We also measured citrate levels, as this metabolite can be generated using acetyl-CoA (produced by the PDH complex) in the TCA; thus, we reasoned that citrate might be reduced following PDH inhibition. The results showed that miR-27b reduced total citrate in ZR75 cells, while inhibition of miR-27b in 4175 increased citrate levels slightly (Fig. 3e). Changes in pyruvate and citrate levels within the medium were not observed (data not shown). However, there are several possible explanations for this. Compared with extracellular lactate, increases in citrate and pyruvate export are a less universal feature of tumor cells [32]. Therefore, while we expected to see increases in medium lactate, we were unsure if and how levels of extracellular citrate and pyruvate would be altered. Of relevance, it has been reported that MCT1—the primary transporter of pyruvate across the plasma membrane—is underexpressed in MCF7 and MDA-MB-231 breast cancer cells compared to non-transformed cells [33], and while loss of MCT1 significantly reduces pyruvate transport, it does not appear to have a consequential effect on lactate export [34]. Additionally, as pyruvate represents a key biosynthetic intermediary, cancer cells would likely benefit from retaining cytosolic pyruvate rather than excreting it [35, 36]. In a similar manner, the regulation of citrate transport may largely account the lack of significant changes in extracellular citrate. [37]. If citrate and pyruvate are not being actively transported across the membrane then any alteration in their production with the cell will not be reflected in the culture medium.

To assess the effects of miR-27b on cell metabolism more globally, we utilized a Seahorse Mitochondrial Stress Test (Agilent Technologies), which allows for the evaluation of the cellular oxygen consumption rate (OCR) and extracellular acidification rate (ECAR). Because mitochondrial oxidative phosphorylation represents the major source of oxygen demand, we could estimate changes in respiration rates by measuring oxygen consumption rates (OCR) using a XFe24 Extracellular Flux Analyzer. The OCR elevated in cells with higher miR-27b expression when mitochondrial oxidative phosphorylation was blocked by FCCP, a compound which uncouples the mitochondrial proton gradient and thereby induces maximum respiration capacity (Fig. 3f, g). In addition, miR-27b overexpression produced a more acidic environment as well, which is characteristic of cells with enhanced glycolytic metabolism (Fig. 3h, i). Notably, this directly correlates with our findings that miR-27b expressing cells also tend to produce excess lactate. Lastly, we directly measured total PDH complex activity level in MCF7 and 4175 cell lysates to quantitate the global capacity of this enzyme in cell lysates. As expected, the overall level of PDH activity in each sample showed an inverse correlation with miR-27b (Fig. 3j).

### Targeting of PDHX specifically is responsible for the metabolic changes resulting from miR-27b overexpression

**Fig. 4:**
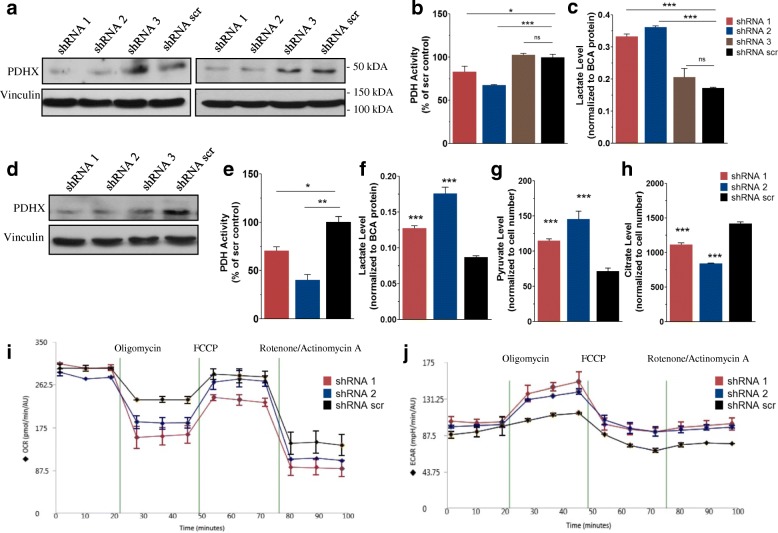
PDHX shRNA recapitulates the effects of miR-27b overexpression. **a** PDHX protein expression in MCF7 (left) and HEK-293 T cells (right) is reduced most strongly by shRNA 1 and 2. PDH complex activity (**b**) and DMEM lactate levels (**c**) following transient expression of shRNA vectors targeting PDHX in MCF7 cells. **d** Stable transfection using the same shRNA expression vectors was carried in MCF7 cells, which were similarly assessed for changes to PDHX protein. These shRNA cells were assessed for changes in PDH activity (**e**), lactate (**f**), pyruvate (**g**) and citrate (**h**) levels. Seahorse mitochondrial stress test quantitating OCR (**i**) and ECAR (J) of MCF7 PDHX KD and control cells

Importantly, it has previously been shown that miR-27b suppresses several other targets. Thus, it cannot be safely assumed that the above findings are due solely to miR-27b’s targeting of PDHX and not an artifact of its inhibition of other known (or unknown) targets. To address this concern, we sought to knockdown PDHX in specific fashion and observe the effects. We obtained shRNA constructs targeting PDHX (Applied Biological Materials) to confirm that miR-27b’s targeting of PDHX alone is necessary and sufficient to reproduce the effects of miR-27b overexpression in cell culture models. We transiently transfected these shRNA constructs into MCF7 and HEK-293 T cells and assessed PDHX protein levels. Two of the shRNAs (shRNA 1 and 2) led to notable reductions in PDHX protein level while shRNA 3 did not alter PDHX in significant fashion compared to the control, (Fig. 4a). Accordingly, we expected the total PDH activity level to fall resulting from the loss of PDHX in shRNA transfected cells. Consistent with the western blot findings, shRNAs 1 and 2 led to decreased PDH activity levels, while shRNA 3 showed no difference from the scr control (Fig. 4b). The medium from these cells was used for evaluating lactate levels; the results correlated as expected with the protein and PDH activity data (Fig. 4c).

Although the transfection efficiencies for the above experiments were generally above 70% (assessed by counting the % GFP+ cells using the GFP marker present in the vector) we created stable cell lines expressing the shRNA constructs in order to eliminate any experimental variation that might arise from the transient transfection process. After puromycin selection, the resulting cells were > 95% GFP+. PDHX protein KD was confirmed by immunoblot (Fig. 4d), and PDH activity level of the shRNA 1 and 2 cells displayed a pattern of low PDH activity in cells with PDH knockdown (Fig. 4e). Intracellular lactate, pyruvate, and citrate were also measured in the shRNA cells, which showed findings matching the results of miR-27b expression (Fig. 4f-h).

We once again employed a Seahorse mitochondrial stress test to assess the cellular metabolic phenotypes by calculating OCR and ECAR The basal oxygen consumption rate was unchanged among the cell lines, but following mitochondrial proton gradient uncoupling by FCCP, the OCR was reduced in cells expressing PDHX shRNA (Fig. 4i). In addition, the shRNA cells displayed a higher ECAR than the control cells (Fig. 4j), suggesting that PDHX KD produces a more acidic environment, which again once again, corresponds to our lactate assay findings.

### Inhibition of PDHX by miR-27b augments in cell growth

**Fig. 5:**
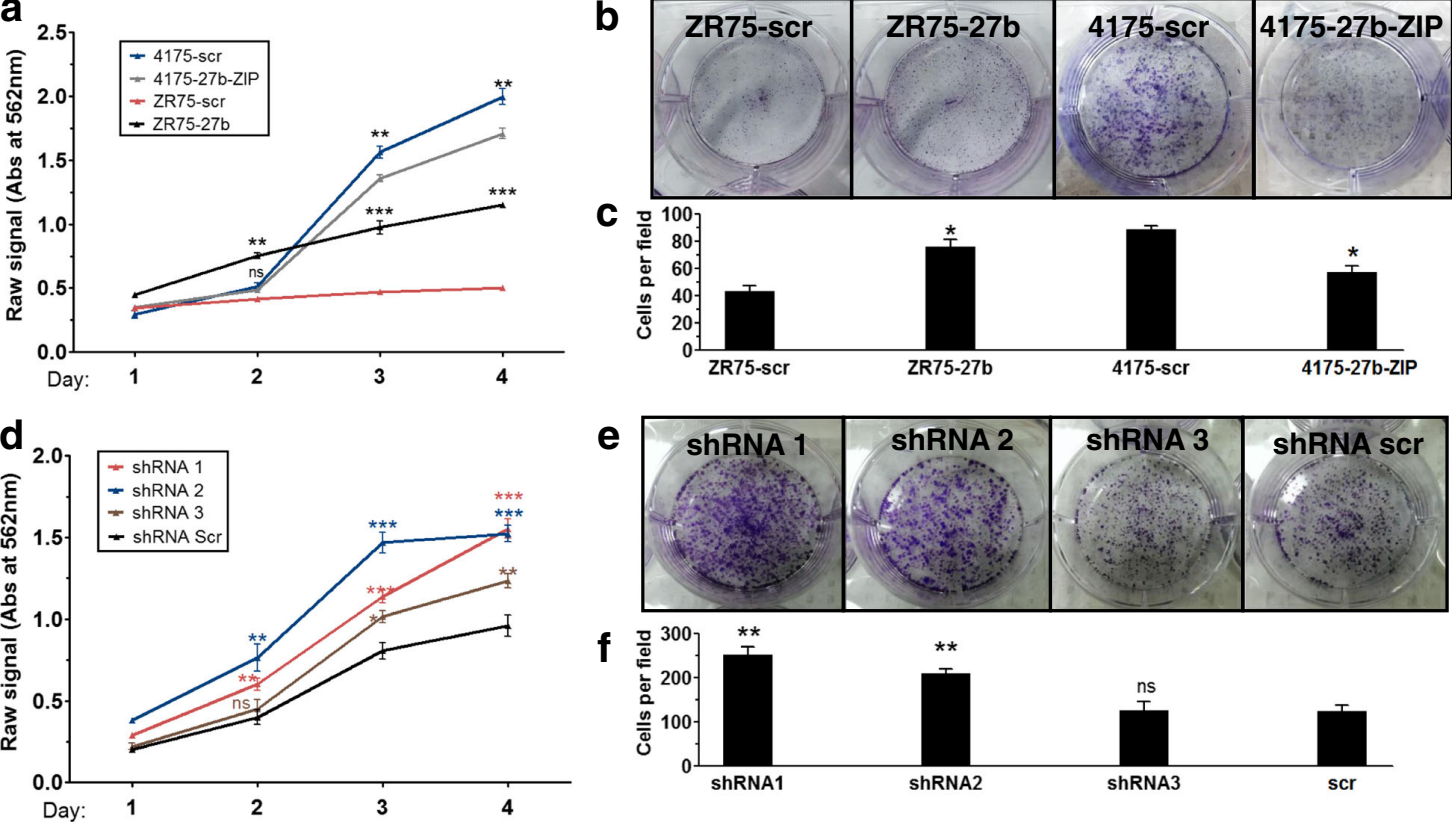
Knockdown of PDHX by miR-27b supports breast cancer cell growth. **a** Time-course MTT assay of ZR75 and 4175 cells expressing miR-27b mimic or miR-27b-ZIP; results shown as raw absorbance at 562 nm, indicating cell number due to growth. **b**, **c** Representative images of a colony formation assay using stable cells stained in crystal violet, with colony counts shown below. **d** MTT assay using cells stably expressing PDHX shRNA; results shown as raw absorbance at 562 nm, indicating cell number due to growth. **e**, **f** Representative images of a colony formation assay using stable cells stained in crystal violet, with colony counts shown below

The suppression of oxidative glucose metabolism is frequently thought to facilitate tumor growth by increasing the supply of glycolytic intermediates available for biosynthesis, and studies have shown that reduced pyruvate oxidation is associated with enhanced cell proliferation [38, 39]. As stated above, the initial step of the oxidative phases of glucose metabolism (oxidative decarboxylation of pyruvate) is catalyzed by the PDH complex. Given our evidence that miR-27b suppresses functioning of this enzyme complex, we wondered whether it may lead to increased proliferative capacity in the cells due to the increased liberation of glycolytic carbon for biosynthesis. Using an MTT assay to measure cell proliferation, we found that low levels of miR-27b corresponded to diminished cell growth in 4175 cells, and higher levels of miR-27b enhanced cell growth in ZR75 cells (Fig. 5a). Similarly, a colony formation assay revealed fewer and smaller-sized colonies following a 2 week incubation in 4175 knockdown cells, whereas more number of cells in miR-27b expressing ZR75 cells. (Figure 5b, c). Importantly, it has previously been shown that miR-27b targets other proteins known to play a role in proliferation, including Nischarin and HIC1. Thus, to assess what effect targeting of PDHX by miR-27b has on cell proliferation independent of its other functions, that we repeated the experiments using the PDHX shRNA cells. The results showed that KD of PDHX with shRNA1 and 2 led to greater cell numbers throughout the course of the experiment compared to scr cells (Fig. 5d). A colony formation assay showed greater colony number in shRNA1 and 2 cells over scr cell control (Fig. 5d, f).

### PDHX expression is reduced in human tumor tissues

**Fig. 6:**
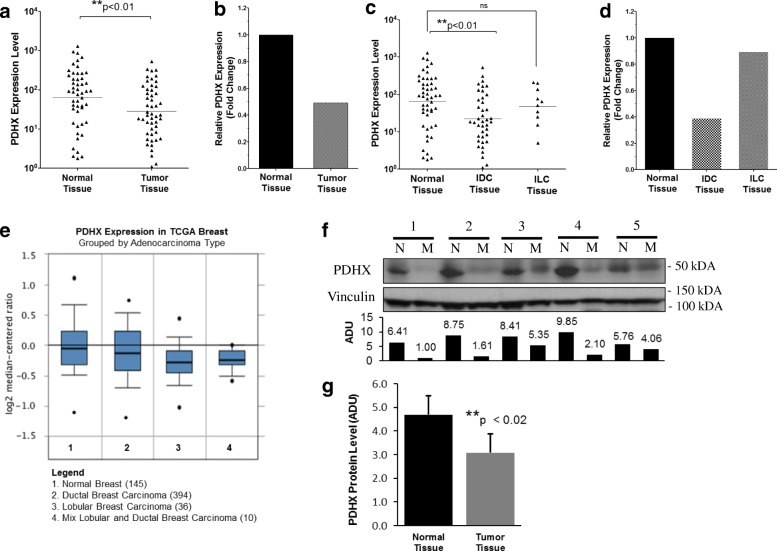
PDHX is inversely correlated with miR-27b in human normal breast and tumor tissues and is associated with improved patient outcomes. **a** Relative PDHX expression level in normal breast (*n* = 55) and breast tumor (*n* = 57) tissues. **b** Fold change in PDHX expression in tissues from panel **a** between normal and cancer tissue sample sets using the ∆∆CT method. **c** Relative PDHX expression level in normal breast compared breast cancer tissues divided by subtype: Invasive ductal carcinoma (IDC) and invasive lobular carcinoma (ILC). **d** Fold change of groups described in panel **c**. **e** Expression of PDHX across multiple breast cancer subtypes in the TCGA database. Number of samples in each category is indicated in parentheses. *p* = 0.03 for ductal breast carcinoma; *p* = 7.15E-4 for lobular breast carcinoma. **f** Representative images of PDHX western blots in tumor samples from five patients. **g** Quantitation of PDHX protein expression from normal (*n* = 10) and tumor (n = 10) patient samples

Given the promising findings we observed in our cell culture models, we next wished to confirm that these observations could be reproduced within human tissues. Our lab has previously amassed a compendium of normal breast and matched tumor tissue specimens. RNA was collected from these samples and analyzed by qRT-PCR., which showed a significant decrease in PDHX expression in the tumor tissues compared to the control tissue group (Fig. 6a, b). This observation fits our hypothesis that PDHX is functionally a tumor suppressor protein which is underexpressed in cancer. Furthermore, the miR-27b expression levels of the samples within this same panel of tissues has previously been assessed by our lab; the published findings reveal that miR-27b expression in the cancer tissues was significantly increased in cancer tissues compared to normal [10]. Thus, the inverse correlation between PDHX and miR-27b observed in cell culture could be reproduced in human breast tissue specimens as well. Interestingly, by subgrouping the tumors according to subtype, we noticed the greatest reduction occurred in invasive ductal tissues (IDC) with a comparatively smaller reduction taking place in invasive lobular tissues (ILC) (Fig. 6c, d). Although the reduction in lobular tissue was not statistically significant, this may be due to the having too few tissue samples necessary to detect a significant reduction, as there were more IDC samples available then ILC samples. In support of this interpretation, evidence from the TCGA database shows PDHX expression is reduced in several breast cancer subtypes, including ductal and luminal subtypes when compared to normal tissue (Fig. 6e). Statistically significant reductions of PDHX in both ductal and lobular subtypes were also observed in data obtained from the Curtis Breast dataset (Additional file 1: Figure S5). To confirm that PDHX protein is also reduced in breast tumors, we used pairwise cancer and normal tissue samples in our collection and probed PDHX protein expression by western blot (Fig. 6f). Quantitation of the protein assay results showed a reduction in PDHX consistent with our previously qPCR findings (Fig. 6g).

**Fig. 7:**
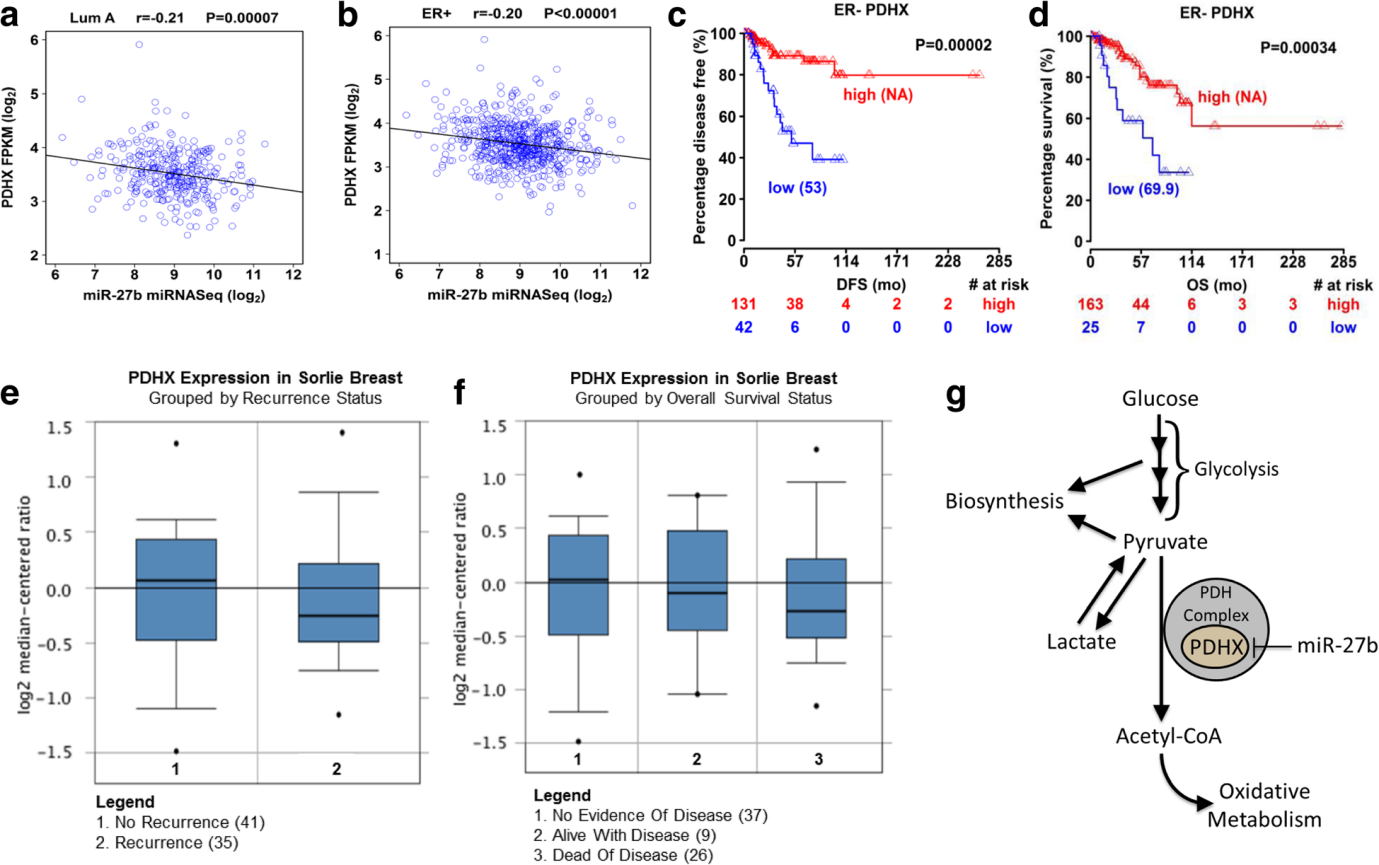
PDHX inversely correlates with miR-27b in human tissues and is associated with improved prognosis and survival. Correlation plots of PDHX and miR-27b TCGA expression data grouped according to luminal A (**a**) and ER+ (**b**) varieties. **b** TCGA data indicating the association between PDHX expression and breast cancer recurrence. Survival curves of breast cancer patients according to PDHX expression status, shown as DFS (**c**) and OS (**d**). Median survival time is shown in brackets. Sorlie Breast Cancer Database statistics showing the association of PDHX with recurrence (**e**) and survival status in breast cancer patients (**f**). **g** Schematic illustrating the miR-27b/PDHX regulatory axis and its functions in regulating cancer cell energy metabolism

To evaluate the correlation of PDHX with miR-27b using tissue expression data beyond that from our own lab, we devised an approach to compare PDHX and miR-27b expression by utilized TCGA expression data for PDHX and miR-27b and performed a correlation plot in luminal A and ER+ BC types (Fig. 7a, b; Additional file 3: Table S2). As seen in our analysis of breast tumors, the expression of PDHX and miR-27b displayed a negative correlation. For additional confirmation, we collected data from the Broad Institute’s GTEx portal to browse RNA-seq data for genes of interest across a panel of 52 human tissue types. All else being equal, tissues with high levels of miR-27b presumably will have low levels of PDHX and vice versa., As anticipated, by graphing mean expression of PDHX and miR-27b in a XY correlation plot we found a significant negative correlation across the dataset (Additional file 1: Figure S6a). This inverse expression pattern can be visualized by graphically comparing the RPKM expression values for PDHX across the entire tissue panel with those of miR-27b, which shows that, in general, tissues with higher PDHX expression tended to have lower miR-27b expression and vice versa (Additional file 1: Figure S7). We repeated this analysis using expression data for C9orf3, the host gene of the miRNA cluster containing miR-27b Presuming that enhanced expression of the C9orf3 parent gene leads to increase in the miRNAs it contains, we plotted C9orf3 expression against PDHX across the tissue panel as before; This too yielded a negative correlation (Additional file 1: Figure S6b). The presumption that greater C9orf3 levels results in greater miR-27b levels was supported by comparing these two genes in an XY plot as well, which showed a positive and statistically significant correlation (Additional file 1: Figure S6c).

To evaluate the role of PDHX in patient outcomes, we performed a Cox regression to evaluate survival in breast cancer patients using TCGA data (Additional file 3: Table S2). We previously showed that elevated miR-27b has detrimental effect on survival in breast cancer patients [9]. To supplement this, we wish to see if PDHX expression had the opposite effect on patient survival. Plots were constructed for PDHX in breast cancer showing Distant-metastasis-free survival (Fig. 7c) and reoccurrence-free survival (Fig. 7d). Both showed a PDHX had a positive effect on survival in ER- patients. The number of patients at risk in low and high mRNA groups at different time points are presented at the bottom of the graph. Median survival months in each group are shown in brackets.

Lastly, to obtain insight concerning how reduced PDHX expression impacts prognosis, we consulted the Sorlie Breast Cancer database to asses for any association of tumor PDHX expression with cancer recurrence and patient surficial status. Of the 71 patients for whom this data was available, the findings suggest that patients with tumors expressing higher levels of PDHX were less likely to have recurrent disease compared to those with lower PDHX levels (Fig. 7e). Similarly, patients who succumbed to their disease displayed lower expression of PDHX than patients alive with disease; PDHX expression was the most increased among patients showing no evidence of disease (Fig. 7f).

## Discussion

As previously outlined, miR-27b has a multifaceted roll in BC by enhancing several processes during initiation and progression of the disease. These include cell migration, invasion, metastasis, and proliferation. However, the precise role of miR-27b in cancer appears to be more nuanced than simply promoting oncogenic processes unilaterally, and tumor suppressive functions have also been attributed to miR-27b as well [40]. However, the existence of differing studies describing seemingly conflicting roles for a miRNA is not uncommon in published literature, illustrating the need for continued investigation to bring clarity to the field. In general, miRNAs are able to target numerous different transcripts; thus, we wished to identify other mRNAs regulated by miR-27b.

It is well known that the rapid pace of cell division intrinsic to neoplasia requires that considerable resources be dedicated to supplying substrates needed for biosynthesis. The intermediates and products of glycolysis represent a key source for this. For example, glycolytic intermediates can be used by the cell to synthesize nucleotides, certain lipids and several amino acids [41–43]. The demand for such substrates has been cited as a major reason accounting for why Warburg metabolism is beneficial to proliferating cancer cells: reducing oxidation of carbohydrates into CO_2_ liberates reduced carbon skeletons which presumably could be better used for biosynthetic purposes than energy production [44]. In addition, by fermenting pyruvate into lactate, the cell can maintain NAD^+^/NADH balance needed to allow for sustained and rapid glycolytic flux and incorporation of glycolytic intermediate into biomass [45].

While a great deal of thought and investigatory rigor has been directed toward substantiating this interpretation, it remains inconclusive whether this explanation fully accounts for the how and why Warburg metabolism directly benefits tumor cells [46]. An alternative explanation recently described proposes that the advantage conferred by aerobic glycolysis arises from the enrichment of cellular glycolytic metabolites, which themselves may be oncogenic by nature [47]. In their study, Peeters et al. observed that the Fructose-1,6-bisphosphate (a key intermediate of glycolysis) is able to activate Ras signaling allosterically, thereby promoting the oncogenic signaling pathways controlled by Ras. Regardless of which explanation is more correct (they are not mutually exclusive), the role which miR-27b plays by inhibiting the PDH complex is applicable in both cases: the accumulation of glycolytic intermediates upstream of the PDH complex is a shared feature of each, and thus would both benefit from PDHX suppression.

In considering the results described here, we propose a mechanism by miR-27b functions to promote breast cancer by deregulating metabolism (Fig. 7g). Through first overexpressing miR-27b, cancer cells are able to inhibit PDHX expression and subsequently function of the PDH complex itself. As a consequence, pyruvate accumulates upstream and is instead shunted into lactate production to enable continued glycolytic flux, or made available to the cell for biosynthetic purposes. The loss of PDH function also leads to reduced cellular respiration, a feature indicating the cancer cell’s shift toward greater dependence on glycolysis. This aligns with previous studies indicating that impaired function of the PDH complex triggers a malignant metabolic phenotype in cancer [18]. This change, together with the increased lactate production, is a distinctive property of Warburg metabolism This metabolic reconfiguration is credited with supporting greater cancer growth [46], through one or more of the hypotheses currently proposed in the field (described in the previous paragraph).

Interestingly, miR-27b appears to promote oncogenic processes via both direct and supporting manners in tandem. For example, miR-27b directly enhances proliferation by targeting of HIC1; simultaneously, however it also indirectly helps facilitate proliferation by reprogramming cell metabolism (via suppression of PDHX) to better align with the metabolic configuration that proliferating cells demand [11]. This is analogous to previous findings showing that it promotes metastasis directly by targeting PSAP, while in parallel also supporting it indirectly by enhancing cell migration/invasion, processes which are generally understood to be a prerequisite for metastasis [12].

Making significant improvements in BC treatment necessitates the willingness to investigate novel and unconventional approaches to therapy. The urgency of improving treatment is highlighted by the heavy disease burden of BC in woman, among whom it remains the leading cause of cancer morbidity and second leading cause of cancer mortality in the US [48]. The growing evidence that altered metabolism is a fundamental component of tumor makeup raises the distinct possibility that future treatments exploiting this feature could prove highly effective [49]. Indeed, its importance in the pathogenesis of cancer has culminated in its recent addition to the list of cancer hallmarks by Hanahan and Weinberg [15]. Though limited, there is some evidence that drugs with metabolic activity have therapeutic utility in cancer. For example, clinical studies report that metformin use is associated with a reduced breast cancer risk in diabetic patients using it to control hyperglycemia [50, 51].

The prolific and promiscuous nature of miRNA regulation of many targets makes them appealing subjects of therapeutic interest. Rather than inhibiting a solitary neoplastic process—an obstacle which cancer cells can frequently circumvent—the blockade of numerous processes simultaneously dramatically reduces the chance that a cancer cell can adapt and subsist. However, this feature of miRNA regulation is two-faced, that is, the non-specific nature of miRNA regulation can result in unintended and unwanted interactions with targets not involved in the cancer process. Thus, from a therapeutic prospective, this feature of miRNA is both its greatest advantage and its chief limitation. Nonetheless, it is not unprecedented for therapeutics with broad effects to be useful (for example, proteasome inhibitors have proven to be therapeutic in treating certain cancers). Indeed, miR-based therapeutics have now undergone phase 1 clinical trials [52].

## Conclusions

Ultimately, miRNA-based strategies could be employed as a practical vehicle through which to target tumor metabolism. To advance this objective, we reveal PDHX to be a target of miR-27b in breast cancer, and characterize the specific metabolic consequences of this dysregulated interaction. Identifying the specific miRNA-mRNA pairs altered in cancer will help enable future efforts to design treatments that target and reverse the specific metabolic alterations underlying a patient’s cancer, potentially resulting in the development of a novel and effective class of cancer therapeutics.

## Additional files

Additional file 1: **Figure S1.** 3’UTRs of PLK2 and PPP1CCC are targeted by miR-27b. Luciferase assays showing the change in luminescence following miR-27b transient transfection verse control of the genes PLK2 (A) and PPP1CCC (B). The putative binding sites are indicated in the boxes below. A cut-off was set for each program giving a binary prediction indicated as 1 or 0. These were tallied and those targets predicted by the most algorithms were considered the best potential targets. **Figure S2.** Luciferase assays for 3’-UTRs not targeted by miR-27b. While prediction algorithms indicated these seven would be good candidates for miR-27b targeting, the predictions could not be validated experimentally. **Figure S3.** Scherf Cell line database evaluation of the expression of PDHX in 11 different types of cancer. Data was accessed using Oncomine platform. **Figure S4.** PDHX expression across a panel of cancer types using the BioExpress gene expression database. **Figure S5.** PDHX expression according to breast adenocarcinoma subtype within the Curtis Breast Statistics dataset. Data was accessed using Oncomine platform. For the Invasive Ductal Breast Carcinoma, *p* = 6.0E-4. For Invasive Lobular Breast Carcinoma subtype, *p* = 5.2E-8. The number of patient samples in each category is indicated in parentheses. **Figure S6.** XY correlation plots of miR-27b with PDHX (A), C9orf3 with PDHX (B) and C9orf3 with miR-27b (C) by RNA-seq across a panel of 52 tissue types retrieved online from the GTEx database. **Figure S7.** Graphical representations of the GTEx RNA-seq expression data of miR-27b, PDHX, and C9orf3 across the panel of 52 human tissue types. (PDF 623 kb)

Additional file 2: **Table S1.** Results of composite miRWalk target prediction. The top 30 are shown. Of these, PPP1CC, CDH11PLK2, JMJD1C, FN1, IRS1, NCOA7, YPEL3, MAP2K4 and PDHX were chosen for validation by luciferase assay. A cut-off was set for each program giving a binary prediction indicated as 1 or 0. These were tallied and those targets predicted by the most algorithms were considered the best potential targets. Raw data used for the survival analysis are included in Additional file 3: Table S2. (XLSX 9 kb)

Additional file 3: **Table S2.** Raw TCGA data used for survival analysis. Patient clinical information was downloaded for the TCGA patients with breast invasive carcinoma (BRCA) from cBioPortal and PDHX gene expression quantification data was downloaded from Genomic Data Commons Data Portal. (XLSX 104 kb)
