# Supplementary material for: Suppression of PDHX by microRNA-27b deregulates cell metabolism and promotes growth in breast cancer
Source: Mol Cancer. 2018 Jul 16;17:100. doi: 10.1186/s12943-018-0851-8 (PMC6048708; doi:10.1186/s12943-018-0851-8)

Supplementary Figure 1

A

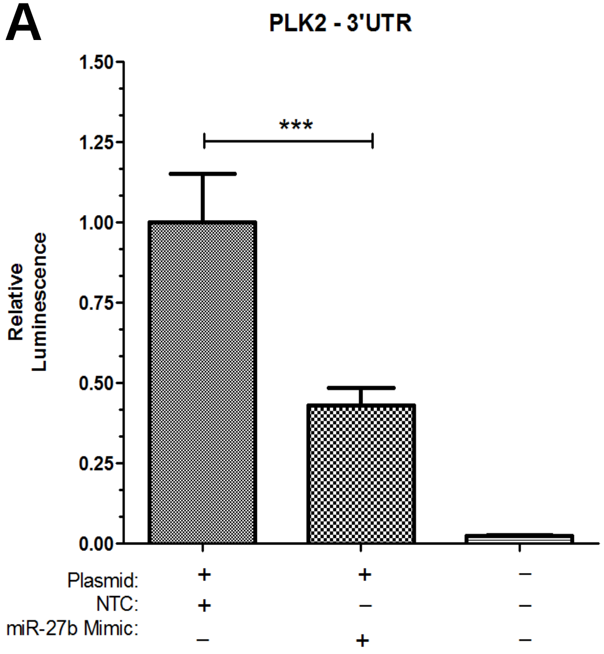

B

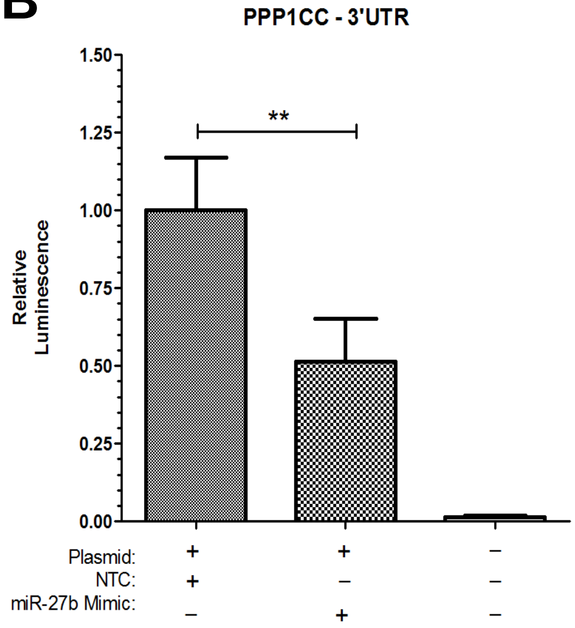

	predicted consequential pairing of target region (top) and miRNA (bottom)	seed match
Position 40-46 of PLK2 3' UTR<br>hsa-miR-27b	5' ...GGACUCCUCUUUUCCACUGUGAG...<br>||| ||||||| 3' CGUCUUGAAUCGGUGACACUU	7mer-m8
Position 320-327 of PLK2 3' UTR<br>hsa-miR-27b	5' ...CCAGAGGACUUUGGAACUGUGAA...<br>|||| ||||||| 3' CGUCUUGAAUCGGUGACACUU	8mer
Position 410-417 of PLK2 3' UTR<br>hsa-miR-27b	5' ...GCUUUUGGCUGCGUAACUGUGAA...<br>||||||| 3' CGUCUUGAAUCGGUGACACUU	8mer

	predicted consequential pairing of target region (top) and miRNA (bottom)	seed match
Position 434-441 of PPP1CC 3' UTR<br>hsa-miR-27b	5' ...CUGUUGAUACAAACCACUGUGAA...<br>||||||| 3' CGUCUUGAAUCGGUGACACUU	8mer

Supplementary Figure 2

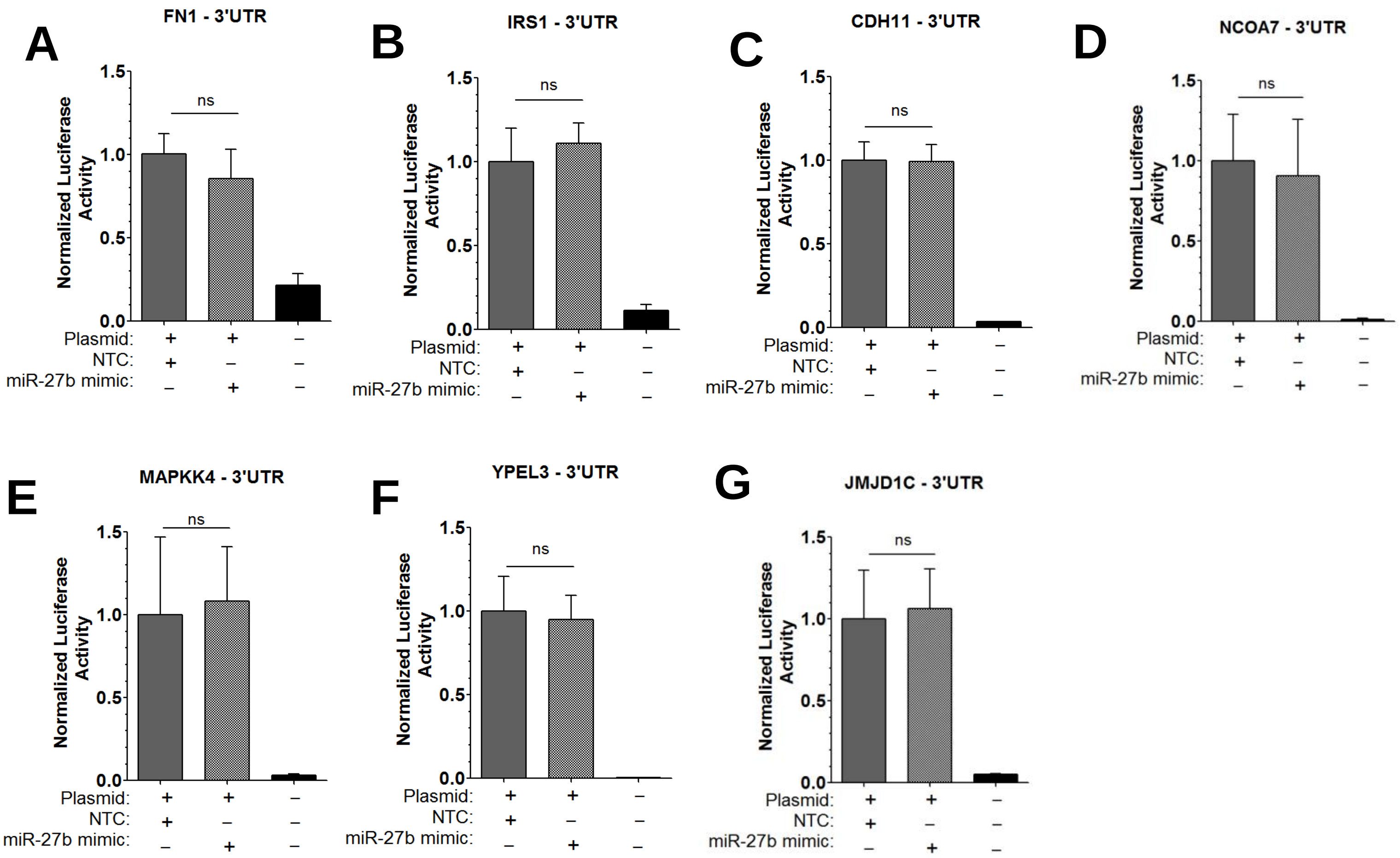

Supplementary Figure 3

Scherf CellLine Statistics

Under-expression Gene Rank: 151 (in top 3%)

Reporter: IMAGE:363410 ▼

P-value: 0.007

t-Test: -3.431

Fold Change: -1.232

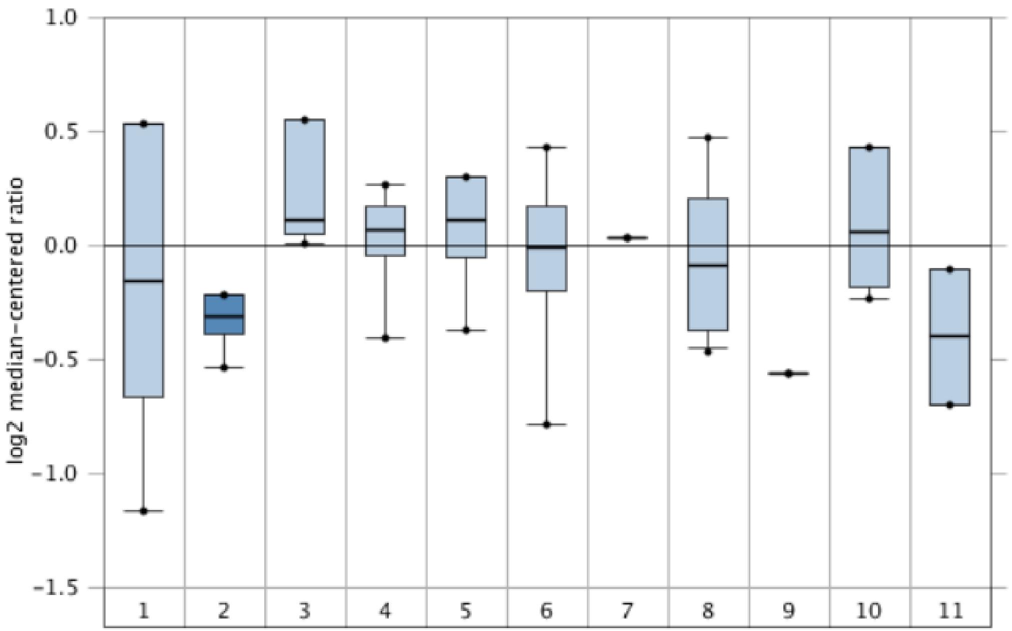

Legend

1. Brain and CNS Cancer (6)
2. Breast Cancer (4)
3. Colorectal Cancer (7)
4. Kidney Cancer (8)
5. Leukemia (4)
6. Lung Cancer (9)
7. Lymphoma (1)
8. Melanoma (10)
9. Myeloma (1)
10. Ovarian Cancer (7)
11. Prostate Cancer (2)

Supplementary Figure 4

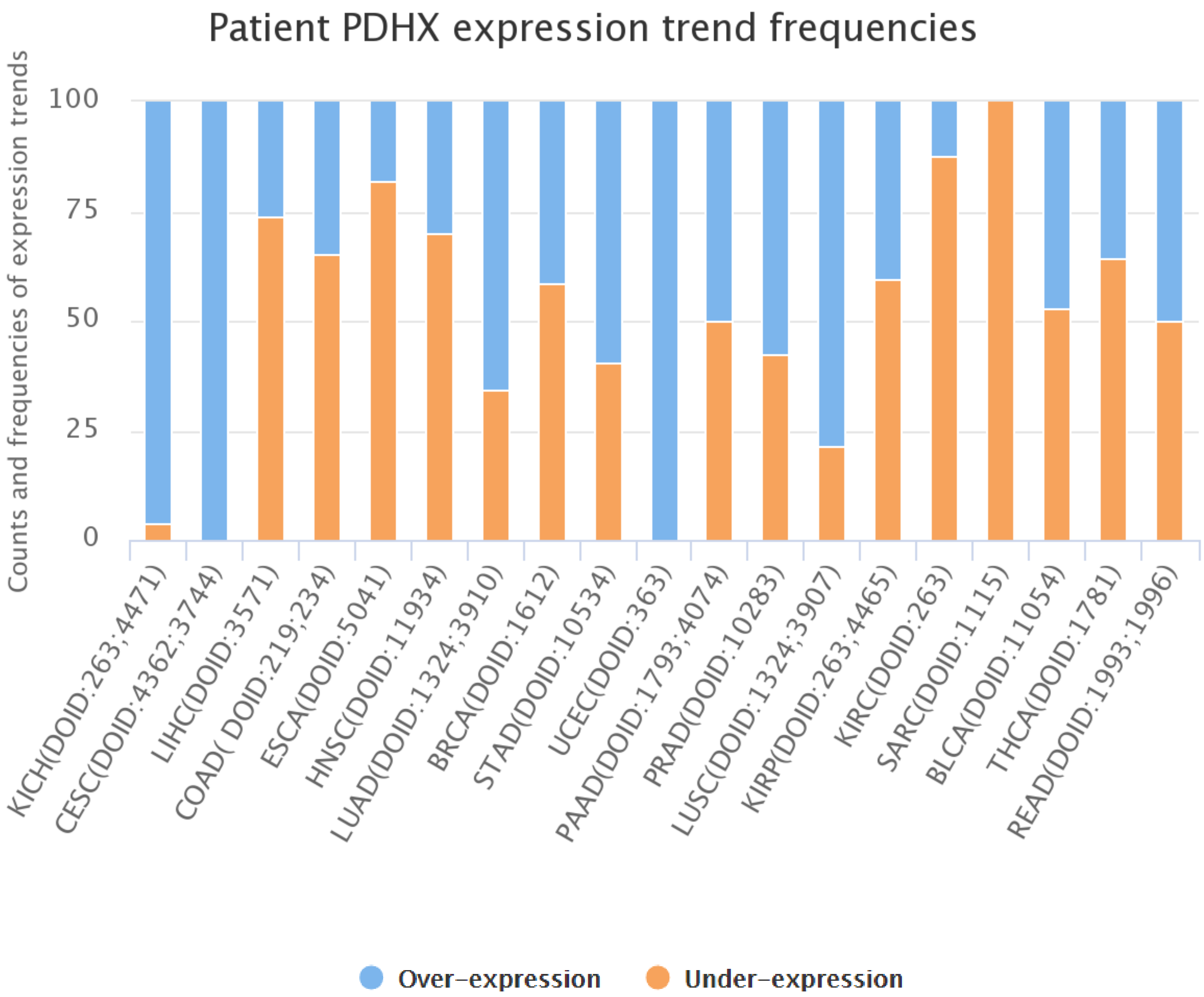

Supplementary Figure 5

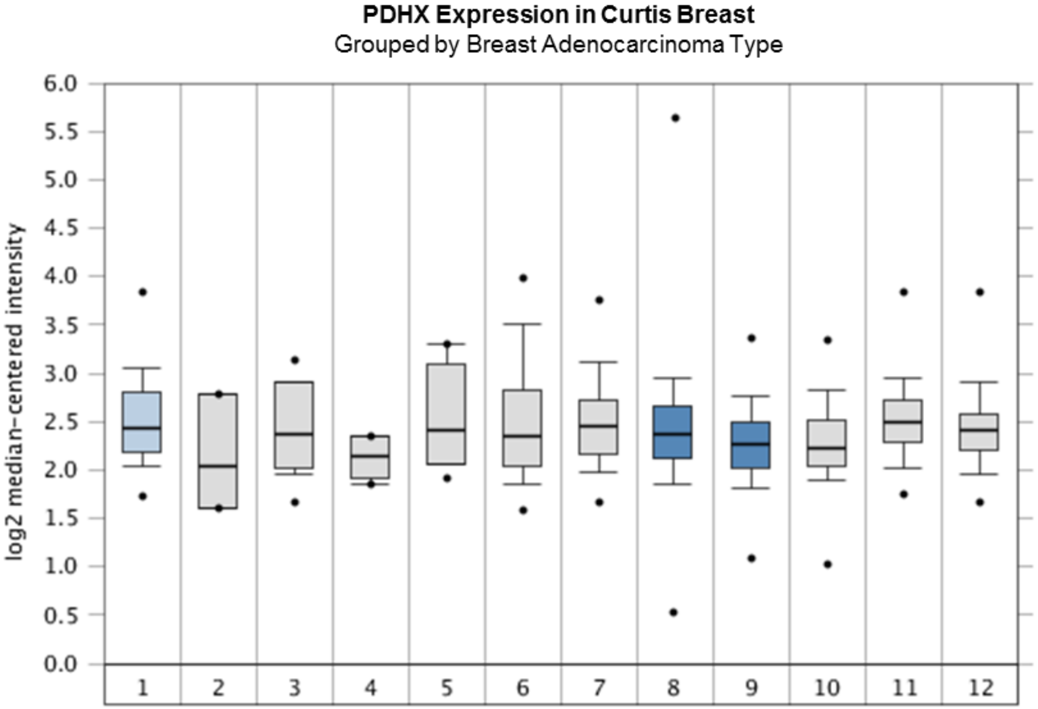

Legend

1. Breast (144)
2. Benign Breast Neoplasm (3)
3. Breast Carcinoma (14)
4. Breast Phyllodes Tumor (5)
5. Ductal Breast Carcinoma in Situ (10)
6. Invasive Breast Carcinoma (21)
7. Invasive Ductal and Invasive Lobular Breast Carcinoma (90)
8. Invasive Ductal Breast Carcinoma (1,556)
9. Invasive Lobular Breast Carcinoma (148)
10. Medullary Breast Carcinoma (32)
11. Mucinous Breast Carcinoma (46)
12. Tubular Breast Carcinoma (67)

Supplementary Figure 6

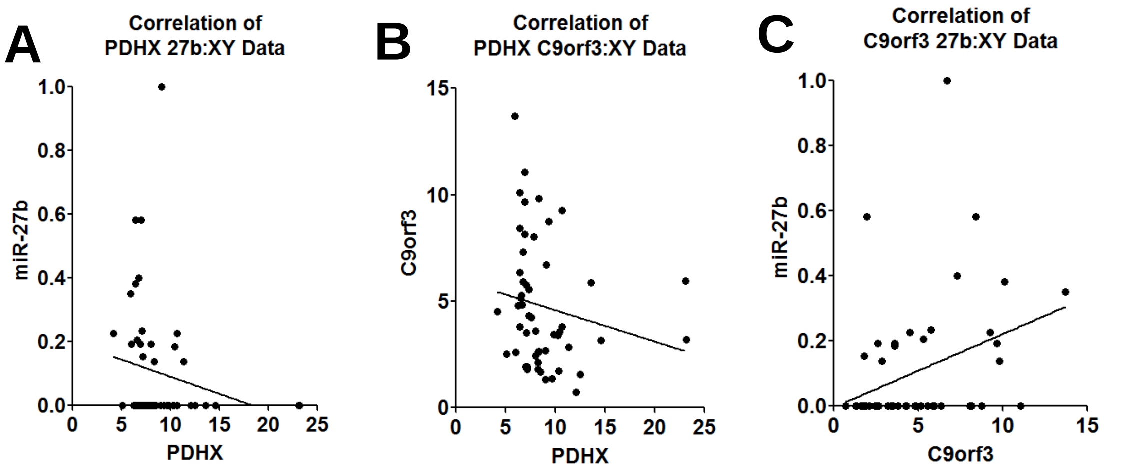

Number of XY Pairs	52
Spearman r	-0.3075
95% confidence interval	-0.5414 to -0.02945
P value (two-tailed)	0.0266
P value summary	*

Number of XY Pairs	52
Spearman r	-0.3400
95% confidence interval	-0.5666 to -0.06571
P value (two-tailed)	0.0136
P value summary	*

Number of XY Pairs	52
Spearman r	0.3704
95% confidence interval	0.1003 to 0.5897
P value (two-tailed)	0.0069
P value summary	**

Supplementary Figure 7

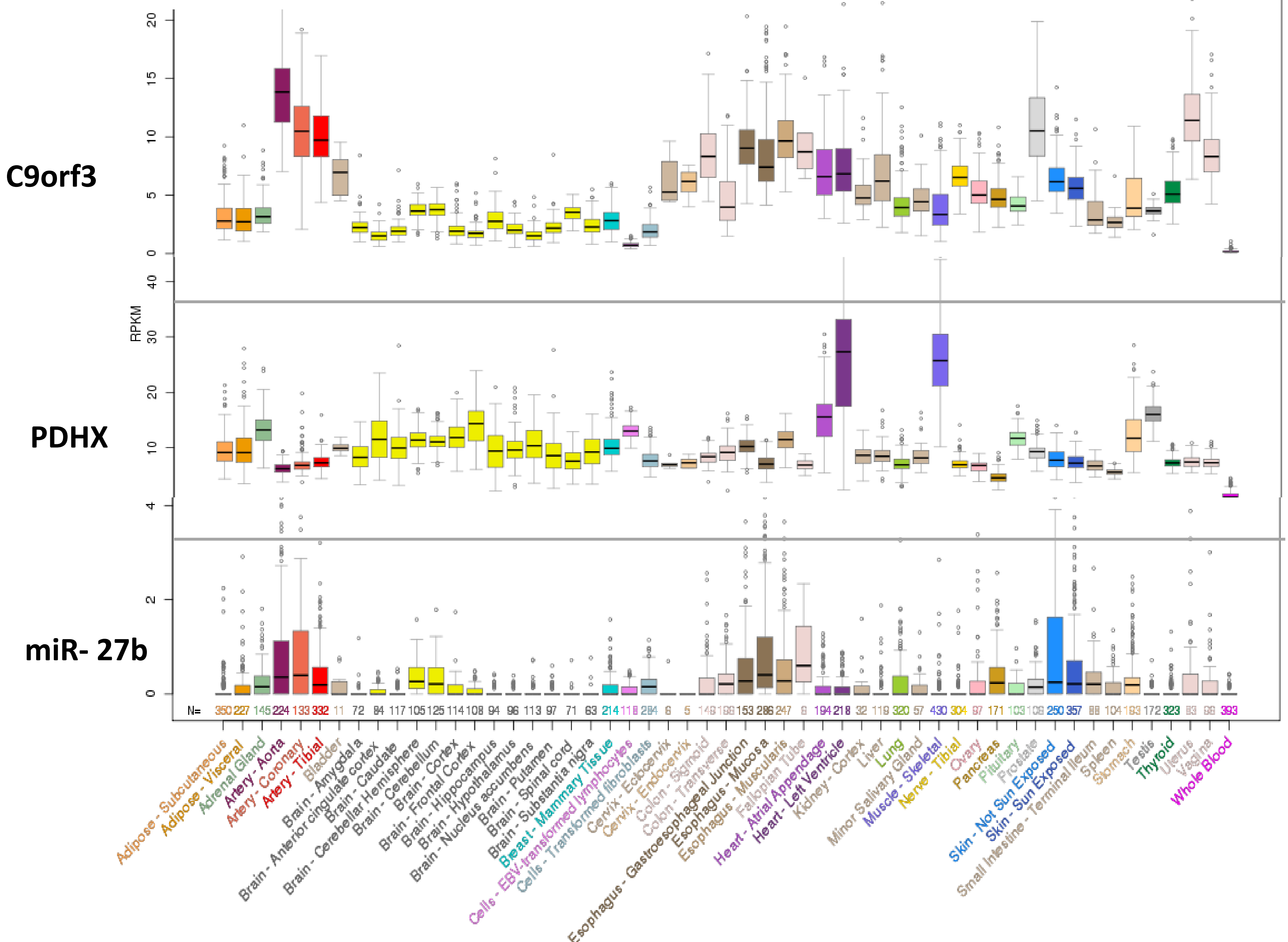

Supplement: Supplementary file 1 — Figure S1. 3’UTRs of PLK2 and PPP1CCC are targeted by miR-27b. Luciferase assays showing the change in luminescence following miR-27b transient transfection verse control of the genes PLK2 (A) and PPP1CCC (B). The putative binding sites are indicated in the boxes below. A cut-off was set for each program giving a binary prediction indicated as 1 or 0. These were tallied and those targets predicted by the most algorithms were considered the best potential targets. Figure S2. Luciferase assays for 3’-UTRs not targeted by miR-27b. While prediction algorithms indicated these seven would be good candidates for miR-27b targeting, the predictions could not be validated experimentally. Figure S3. Scherf Cell line database evaluation of the expression of PDHX in 11 different types of cancer. Data was accessed using Oncomine platform. Figure S4. PDHX expression across a panel of cancer types using the BioExpress gene expression database. Figure S5. PDHX expression according to breast adenocarcinoma subtype within the Curtis Breast Statistics dataset. Data was accessed using Oncomine platform. For the Invasive Ductal Breast Carcinoma, p = 6.0E-4. For Invasive Lobular Breast Carcinoma subtype, p = 5.2E-8. The number of patient samples in each category is indicated in parentheses. Figure S6. XY correlation plots of miR-27b with PDHX (A), C9orf3 with PDHX (B) and C9orf3 with miR-27b (C) by RNA-seq across a panel of 52 tissue types retrieved online from the GTEx database. Figure S7. Graphical representations of the GTEx RNA-seq expression data of miR-27b, PDHX, and C9orf3 across the panel of 52 human tissue types. (PDF 623 kb) [file 12943_2018_851_MOESM1_ESM.pdf]
